# Oncolytic reovirus inhibits angiogenesis through induction of CXCL10/IP-10 and abrogation of HIF activity in soft tissue sarcomas

**DOI:** 10.18632/oncotarget.21423

**Published:** 2017-09-30

**Authors:** Jennifer S. Carew, Claudia M. Espitia, Weiguo Zhao, Monica M. Mita, Alain C. Mita, Steffan T. Nawrocki

**Affiliations:** ^1^ University of Arizona Cancer Center and Department of Medicine, Division of Translational and Regenerative Medicine, University of Arizona, Tucson, AZ, USA; ^2^ Division of Hematology/Oncology, Cancer Therapy and Research Center at The University of Texas Health Science Center at San Antonio, San Antonio, TX, USA; ^3^ Samuel Oschin Comprehensive Cancer Institute, Cedars-Sinai Medical Center, Los Angeles, CA, USA

**Keywords:** reovirus, CXCL10, HIF-1, angiogenesis, reolysin

## Abstract

The tumor-selective viral replication capacity and pro-apoptotic effects of oncolytic reovirus have been reported to be dependent on the presence of an activated RAS pathway in several solid tumor types. However, the mechanisms of selective anticancer efficacy of the reovirus-based formulation for cancer therapy (Reolysin, pelareorep) have not been rigorously studied in soft tissue sarcomas (STS). Here we report that Reolysin triggered a striking induction of the anti-angiogenic chemokine interferon-γ-inducible protein 10 (IP-10)/CXCL10 (CXC chemokine ligand 10) in both wild type and RAS mutant STS cells. Further analysis determined that Reolysin treatment possessed significant anti-angiogenic activity irrespective of RAS status. In addition to CXCL10 induction, Reolysin dramatically downregulated the expression of hypoxia inducible factor (HIF)-1α, HIF-2α and inhibited vascular endothelial growth factor (VEGF) secretion. CXCL10 antagonism significantly diminished the anti-angiogenic effects of Reolysin indicating that it is a key driver of this phenomenon. Xenograft studies demonstrated that Reolysin significantly improved the anticancer activity of the anti-angiogenic agents sunitinib, temsirolimus, and bevacizumab in a manner that was associated with increased CXCL10 levels. This effect was most pronounced following treatment with Reolysin in combination with temsirolimus. Further analysis in additional sarcoma xenograft models confirmed the significant increase in CXCL10 and increased anticancer activity of this combination. Our collective results demonstrate that Reolysin possesses CXCL10-driven anti-angiogenic activity in sarcoma models, which can be harnessed to enhance the anticancer activity of temsirolimus and other agents that target the tumor vasculature.

## INTRODUCTION

Established chemotherapy regimens for advanced sarcoma yield unacceptably low 5-year survival rates and are characterized by significant toxicity that is very difficult for patients to tolerate. The frequent development of drug resistance further underscores the urgent need for more effective and less toxic treatment approaches [1]. Reoviruses are commonly found in the respiratory and gastrointestinal tracts of humans, but are not associated with any diseases and exposure is normally asymptomatic. Recent studies have demonstrated that reoviruses specifically replicate only in certain cancer cell types, such as those with mutant RAS. This led to the development of a reovirus-based formulation for cancer therapy called Reolysin (pelareorep) [2, 3]. Clinical studies have demonstrated that systemic administration of Reolysin has significant anticancer activity and notably, is very well tolerated with minimal unpleasant side effects [4]. However, the mechanism(s) underlying Reolysin’s anticancer effects remain to be fully elucidated and its utility as a novel anti-sarcoma therapy has not been rigorously investigated.

The preferential replication of reovirus in cancer cells with mutant RAS has been attributed to its ability to inhibit double stranded RNA-activated protein kinase (PKR) activity [2, 3]. Activation of RAS inhibits PKR and subsequent eukaryotic initiation factor 2 ɑ-subunit (eif2ɑ) phosphorylation, resulting in an accumulation of viral particles inside cancer cells. However, recent findings demonstrate that reovirus has significant anticancer activity irrespective of RAS status in certain cancer models [5, 6]. This may be attributed to high levels of the reovirus receptor junctional adhesion molecule-A in some cancer types [5, 7]. A better understanding of the mechanisms of reovirus-mediated anticancer efficacy will enable us to optimally position it with rational chemotherapy combinations that maximize its activity.

We investigated the ability of oncolytic reovirus to replicate in sarcoma cell lines with mutant and wild type (WT) RAS status and investigated its anticancer activity in both *in vitro* and *in vivo* models. In agreement with prior reports in various solid tumor models, reovirus replicated much more efficiently in cancer cells with mutant RAS [2, 8]. However, we demonstrate that reovirus treatment strongly upregulated the anti-angiogenic chemokine CXCL10 and decreases HIF activity in sarcoma cells with differing RAS status. Here we report that Reolysin possesses significant *in vivo* anticancer activity in sarcoma models and potently inhibits angiogenesis. In addition, Reolysin treatment augmented the anti-angiogenic effects and anticancer efficacy of approved agents sunitinib, bevacizumab, and temsirolimus. Our data supports further investigation of Reolysin in combination with angiogenesis targeted therapies for the precision treatment of highly vascular tumors where angiogenesis plays an essential role in tumor progression.

## RESULTS

### Reovirus preferentially replicates in *NRAS* mutant sarcoma cells

**Figure 1:**
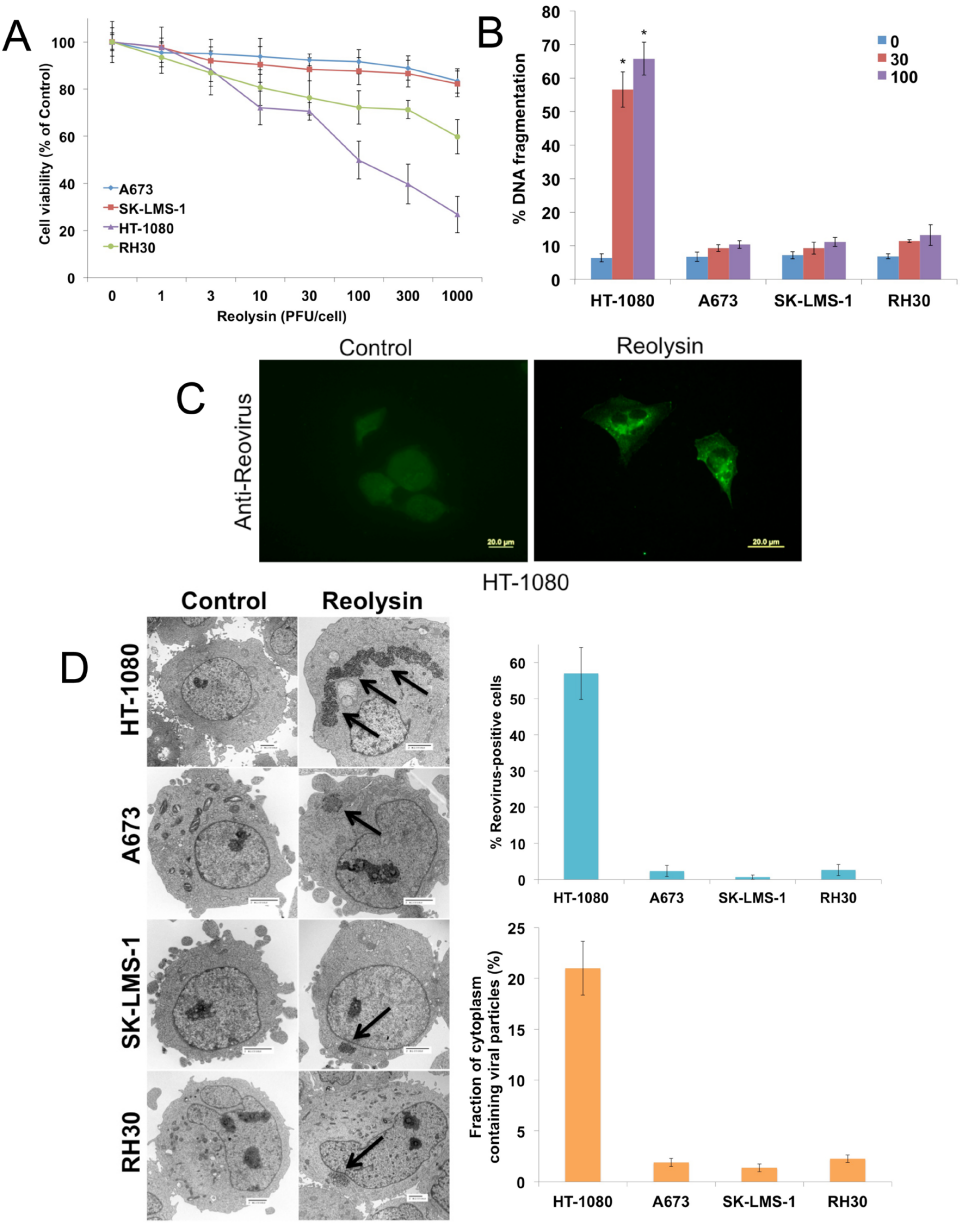
Reovirus preferentially replicates in NRAS-mutant HT-1080 sarcoma cells **(A)** The effects of oncolytic reovirus on sarcoma cells. Sarcoma cell lines were treated for 72 h with the indicated concentrations of Reolysin. Cell viability (based on quantification of mitochondrial metabolism) was determined by MTT assay. Mean ± SD, *n* = 3. **(B)** HT-1080 sarcoma cells are sensitive to Reolysin-mediated cell death. Cells were treated for 48 h with Reolysin and DNA fragmentation was measured by PI-FACS analysis. Mean ± SD, *n* = 3. *Indicates a significant difference compared to Controls, *p* < 0.05. **(C)** Reovirus replicates in HT-1080 cells. Cells were treated with 30 PFU/cell Reolysin for 48 h and stained with an anti-reovirus antibody. Immunocytochemistry reveals significant reovirus replication in infected HT-1080 cells. Reovirus replication was not observed in A673, SK-LMS-1, and RH30 cell lines. **(D)** Quantification of reovirus replication in sarcoma cell lines. Cells were treated with 30 PFU/cell Reolysin for 48 h and reovirus replication was visualized by electron microscopy. Arrows denote reovirus particles. The percentages of reovirus infected cells were manually counted in 3 distinct areas of 100 cells (top). The fraction of cytoplasm containing viral particles was quantified using ImageJ software. (bottom) Mean ± SD, *n* = 3.

Previous studies have reported that reovirus selectively replicates in cells that harbor a *RAS* mutation or an activated RAS pathway through upstream stimulation [2, 9, 10]. We evaluated the activity of Reolysin in sarcoma cell lines with varying *RAS* mutation status (Supplymentary Table 1). The *NRAS* mutant HT-1080 cell line displayed hypersensitivity to Reolysin treatment as demonstrated by a significantly greater reduction in cell viability (Figure 1A) and induction of apoptosis (Figure 1B) compared with the WT RAS cell lines (A673, RH30, and SK-LMS-1). Consistent with the potent anticancer activity of Reolysin that we observed in HT-1080 cells, reovirus was easily detected by immunofluorescence in the cytoplasm of these cells (Figure 1C). Reovirus accumulation was below the level of detection by immunofluorescence in the 3 WT RAS sarcoma cell lines (data not shown). To better quantify reovirus replication in these models, electron microscopy was performed and the percentage of cells positive for reovirus infection and the fraction of cytoplasm containing viral particles were quantified (Figure 1D). As expected, reovirus replication was very prominent in the HT-1080 cells with minimal positive replication detected in the WT RAS sarcoma cell lines.

### Reolysin induces CXCL10 and an IFN response in sarcoma cells

**Figure 2:**
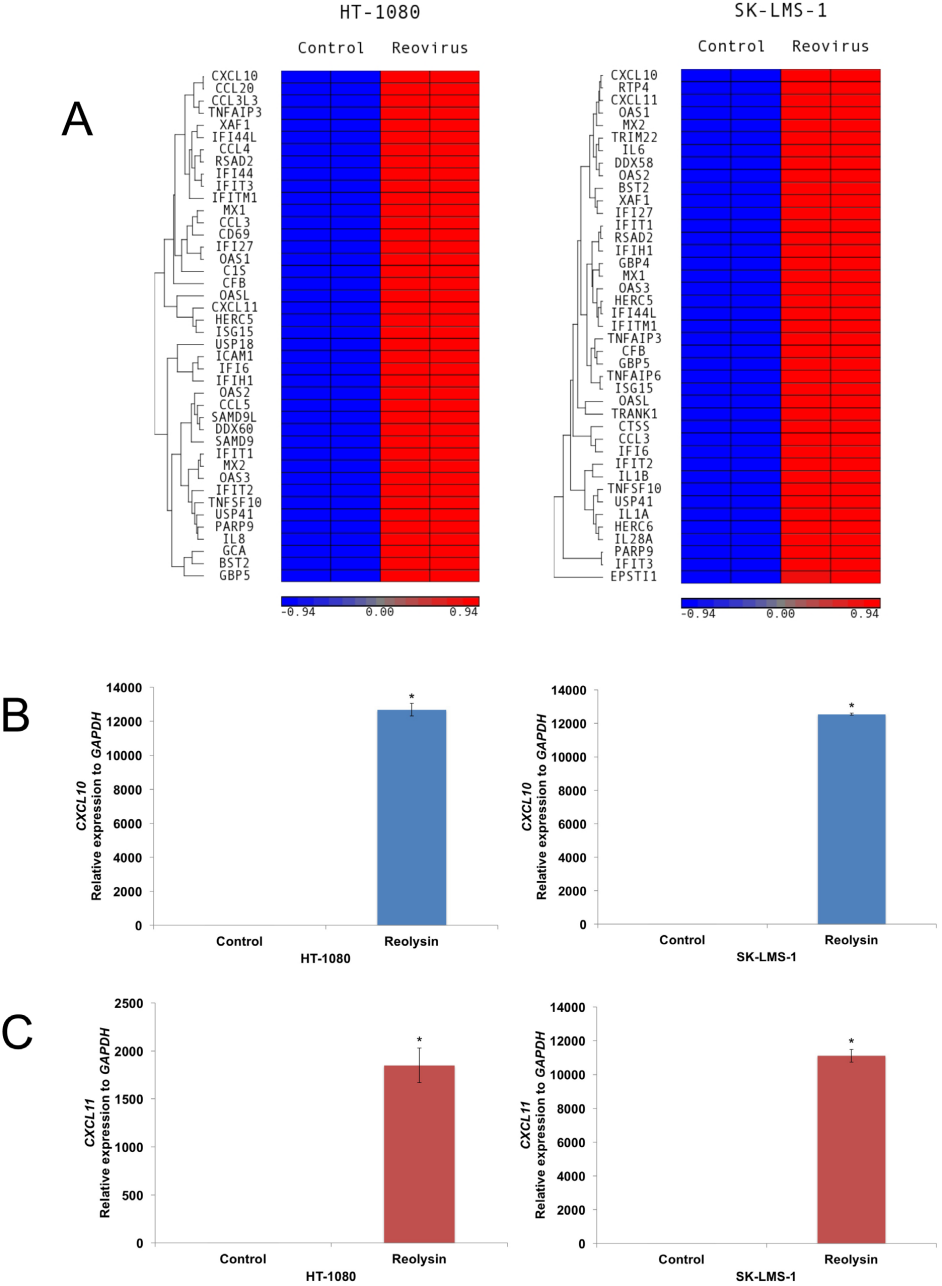
Oncolytic reovirus induces *CXCL10* expression **(A)** Affymetrix expression arrays revealed a significant increase in *CXCL10* in mutant RAS HT-1080 cells and WT RAS SK-LMS-1 cells after 48 h treatment with 30 PFU/cell Reolysin. **(B-C)** qRT-PCR analysis of *CXCL10* and *CXCL11*. Cells were treated with 30 PFU/cell Reolysin for 48 h and then harvested for analysis. Levels of mRNAs were standardized to the expression of *GAPDH*. Mean ± SD, *n* = 3, *Indicates a significant difference from the controls, *p* < 0.05.

**Figure 3:**
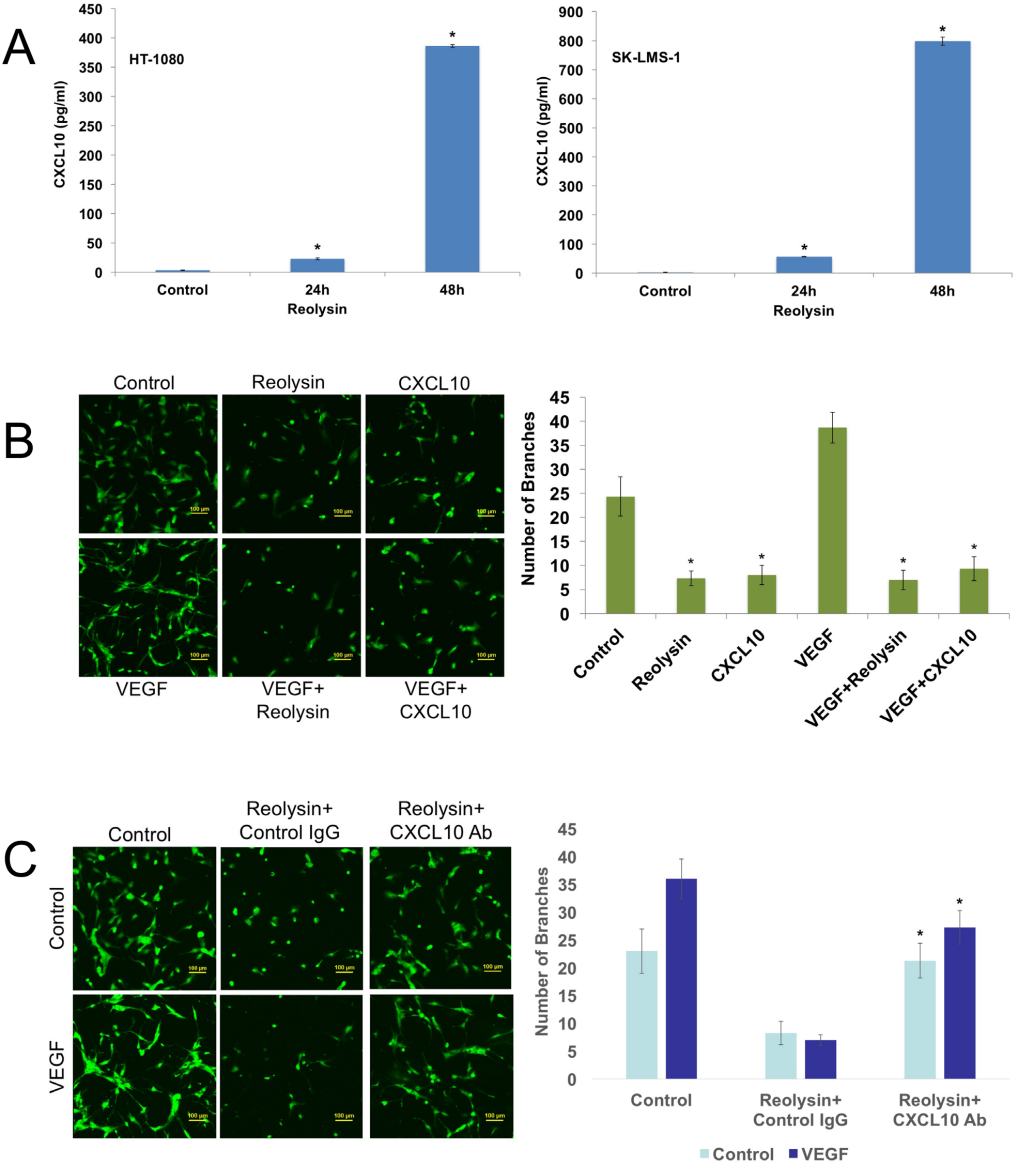
Reolysin induced CXCL10 expression contributes to inhibition of endothelial tube formation **(A)** Reolysin promotes CXCL10 secretion. HT-1080 and SK-LMS-1 cells were treated with 30 PFU/cell Reolysin for 24 and 48 h. CXCL10 expression was determined by ELISA analysis of cell culture media following treatment. **(B)** Reolysin and CXCL10 inhibit HUVEC tube formation. HUVECs were grown in M200 media and incubated with 30 PFU/cell Reolysin or 200 ng/ml recombinant CXCL10 with or without the presence of 100 ng/ml VEGF for 24 h. Tube formation was quantified as described in Materials and Methods. Mean ± SD, *n* = 3, *Indicates a significant difference from Control and VEGF stimulated cells, *p* < 0.05. **(C)** CXCL10 induction significantly contributes to Reolysin-mediated inhibition of HUVEC tube formation. HUVECs were grown in M200 media and incubated with 30 PFU/cell Reolysin or 2 μg/ml anti-CXCL10 antibody with or without the presence of 100 ng/ml VEGF for 24 h. Tube formation was quantified as described in Materials and Methods. Mean ± SD, *n* = 3, *Indicates a significant difference from Reolysin treated cells, *p* < 0.05.

To further characterize the effects of Reolysin on sarcoma cells, we evaluated gene expression changes following reovirus infection in mutant (HT-1080) and WT (SK-LMS-1) *RAS* sarcoma models by microarray analysis (Supplymentary Tables 2 and 3). Not surprisingly, reovirus infection stimulated a strong increase in genes associated with an anti-viral response in both cell lines, including *OAS1*, *OAS2*, and *OAS3* (Figure 2A). Of the upregulated genes, interferon (IFN)-γ-induced protein 10 (IP-10/CXCL10) was dramatically induced in both cell lines. To validate our microarray findings, qRT-PCR analysis confirmed a potent induction in CXCL10 expression and the related gene CXCL11 (Figure 2B and 2C). Furthermore, ELISA assay demonstrated that CXCL10 secretion was significantly increased in sarcoma cells exposed to Reolysin (Figure 3A). While CXCL10 plays a role in immune response and inflammation, it has also been reported to inhibit angiogenesis [11–17]. To investigate the anti-angiogenic potential of Reolysin and CXCL10, we treated HUVEC cells with VEGF in the presence of Reolysin and recombinant CXCL10 and measured capillary tube formation. Reolysin and CXCL10 both significantly inhibited capillary structure formation indicating that Reolysin and CXCL10 can prevent VEGF-induced tube formation in endothelial cells (Figure 3B). We next evaluated whether CXCL10 neutralizing antibody could blunt the anti-angiogenic activity of Reolysin with or without VEGF exposure. Consistent with the role of CXCL10 in reducing angiogenesis, neutralizing CXCL10 decreased the ability of Reolysin to block tube formation in endothelial cells (Figure 3C). Collectively, these data demonstrate that CXCL10 induction is a significant contributor to Reolysin-mediated inhibition of angiogenesis.

### Treatment with Reolysin antagonizes HIF-1ɑ activity and VEGF secretion in sarcoma cells

**Figure 4:**
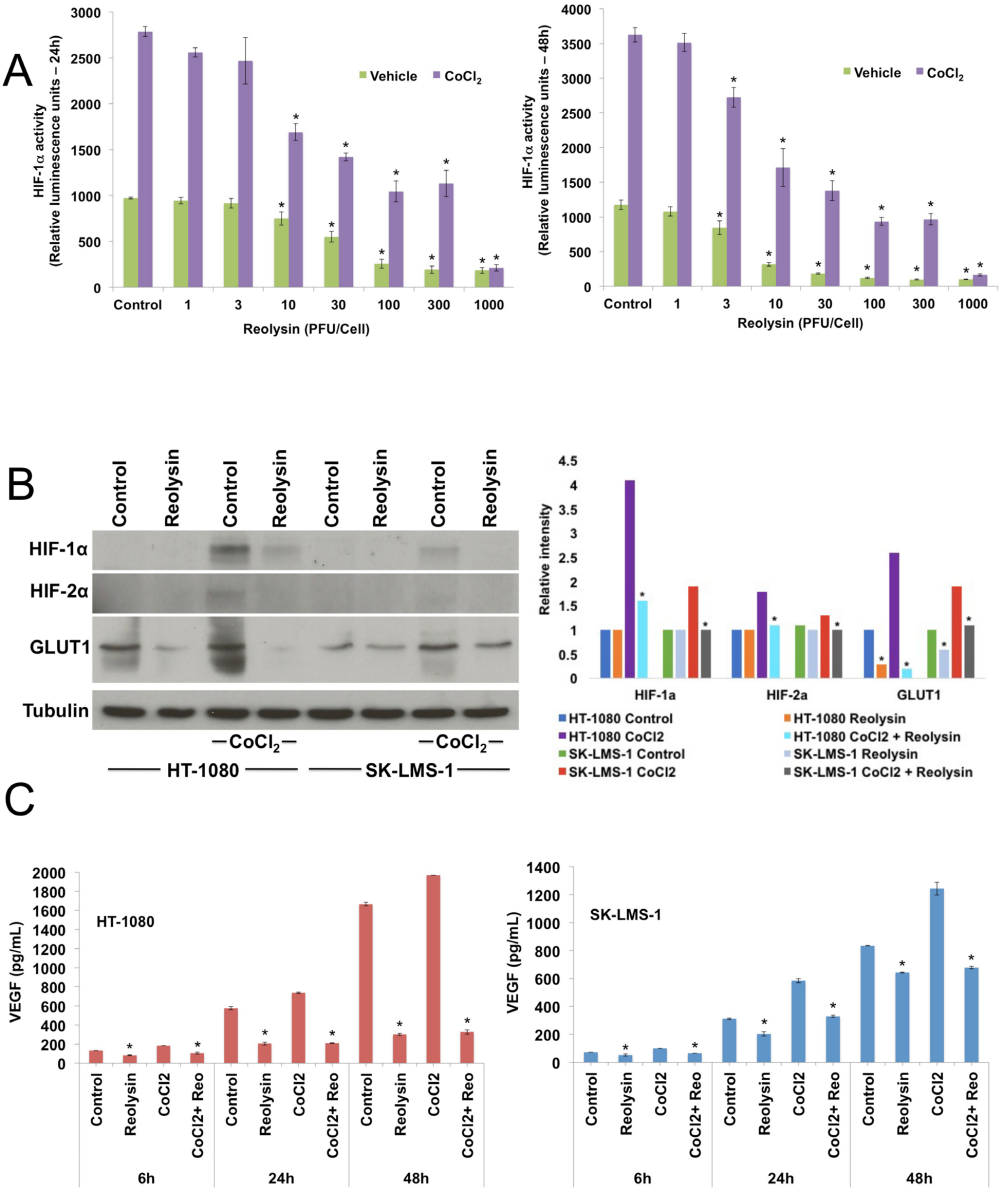
Reolysin decreases HIF expression and reduces VEGF secretion **(A)** Reolysin decreases HIF-1ɑ activity. HCT116 cells stably expressing a HIF-1ɑ luciferase reporter were treated with the indicated concentrations of Reolysin with or without the presence of 100 μM CoCl_2_ for 24 and 48 h. HIF-1ɑ activity was determined by measuring luminescence. Mean ± SD, *n* = 3, *Indicates a significant difference from Vehicle control cells, *p* < 0.05. **(B)** Reolysin decreases the expression of HIF-1ɑ, HIF-2ɑ, and GLUT1. Sarcoma cells were treated with 30 PFU/cell Reolysin with or without the presence of CoCl_2_ for 24 h and protein levels were measured by immunoblotting. Bands were quantified by densitometry. *Indicates a significant difference compared to Control or CoCl_2_ stimulated cells, *p* < 0.05. **(C)** Reolysin reduces VEGF secretion. HT-1080 and SK-LMS-1 cells were treated with 30 PFU/cell Reolysin for 6, 24, and 48 h with or without CoCl_2_. VEGF secretion was measured by ELISA. Mean ± SD, *n* = 3, *Indicates a significant difference from control cells at the same time, *p* < 0.05.

HIF-1ɑ is a key transcription factor involved with adaptation and survival of cells in the hypoxic microenvironment [18, 19]. Since we observed significant inhibition of *in vitro* angiogenesis with Reolysin, we next investigated the effects of reovirus infection on HIF-1ɑ activity. Using a HIF-1ɑ NanLuc reporter cell line, cells were incubated in the presence or absence of cobalt chloride (CoCl_2_) to mimic hypoxia and treated with increasing concentrations of Reolysin for 24 and 48 hours. Reolysin infection alone was sufficient to significantly inhibit basal HIF-1ɑ activity at both time points (Figure 4A). Importantly, we also observed a significant reduction in HIF-1ɑ expression in sarcoma cells lines following hypoxic stimulation with CoCl_2_ (Figure 4B). In addition to direct HIF-1ɑ suppression, we were also able to observe a significant decrease in HIF-2ɑ and the downstream target GLUT1 (Figure 4B). Consistent with the observed decrease in HIF expression, the levels of the well characterized HIF-regulated gene VEGF were also markedly reduced following Reolysin treatment in both HT-1080 and SK-LMS-1 models (Figure 4C).

### Reolysin cooperates with the anti-angiogenic agents temsirolimus, sunitinib, and bevacizumab to stimulate CXCL10 expression and enhance anti-sarcoma activity

**Figure 5:**
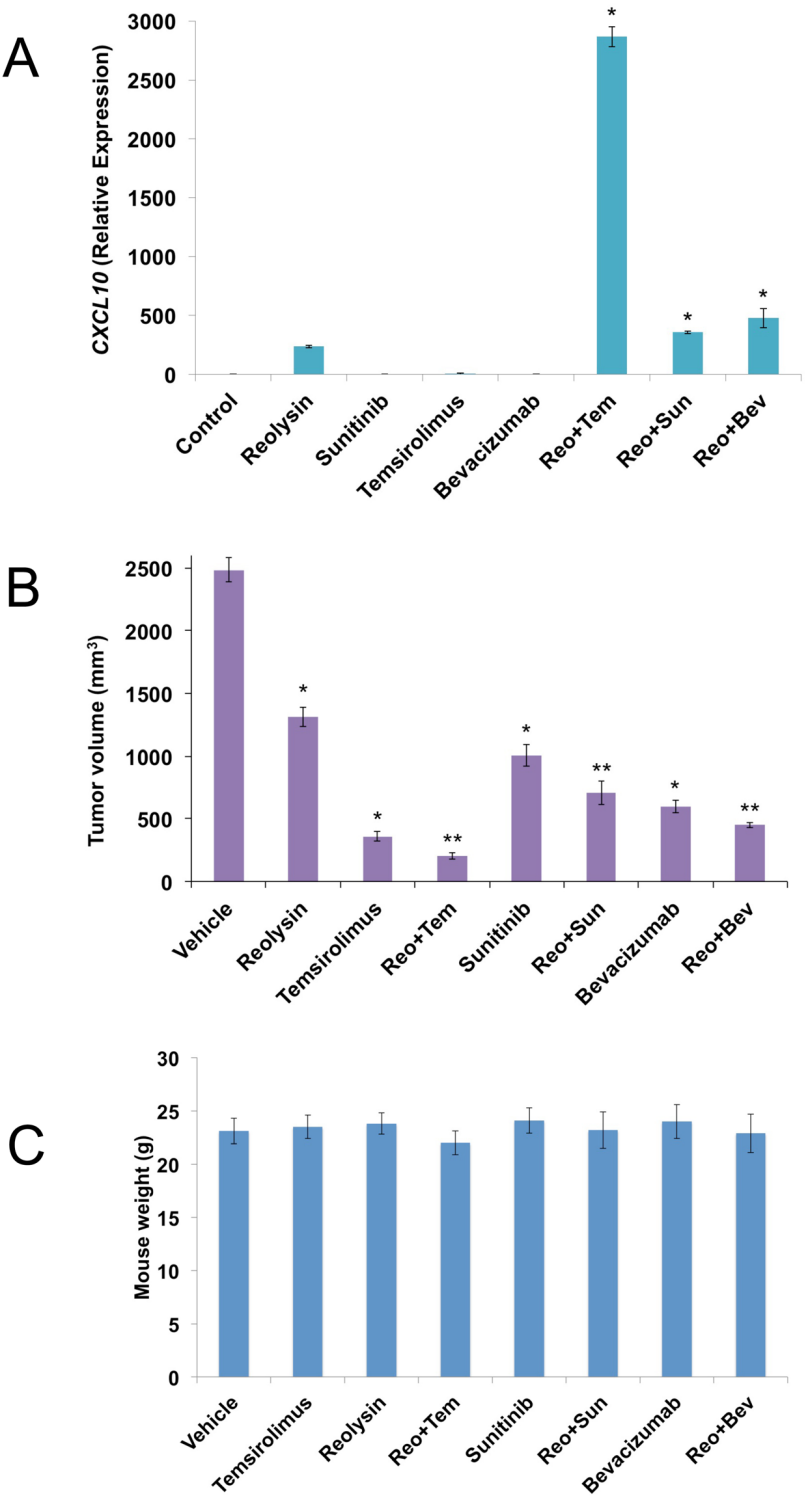
Reolysin augments the anticancer activity of temsirolimus, sunitinib, and bevacizumab **(A)** Anti-angiogenic agents increase Reolysin-induced CXCL10 levels. SK-LMS-1 cells were treated with 30 PFU/cell Reolysin, 5 μM sunitinib, 50 nM temsirolimus, 1 mg/ml bevacizumab, and combinations for 24 h. CXCL10 levels were measured by qRT-PCR, Levels of mRNAs were standardized to the expression of *GAPDH*. Mean ± SD, n = 3, *Indicates a significant difference from Control and single agent treatments, *p* < 0.05. **(B)** SK-LMS-1 cells (1 x 10^7^/mouse) were injected into the flanks of nude mice. When tumors reached approximately 150 mm^3^ in size, mice were randomized into groups and treated with 5 x 10^8^ TCID_50_ Reolysin Q7D, 5 mg/kg temsirolimus QD, 40 mg/kg sunitinib QD, 10 mg/kg bevacizumab Q3D, or combinations as described in Materials and Methods. After 3 weeks of treatment, tumor volume was quantified. Mean ± SEM, *n* = 10. *Indicates a significant difference compared to vehicle or **either single agent treatment, *p* < 0.05. **(C)** Reolysin and drug combinations are well tolerated in mice. Animal body weight was determined at the end of the study (Day 21) to quantify drug-induced weight loss. Mean ± SD, *n* = 10.

**Figure 6:**
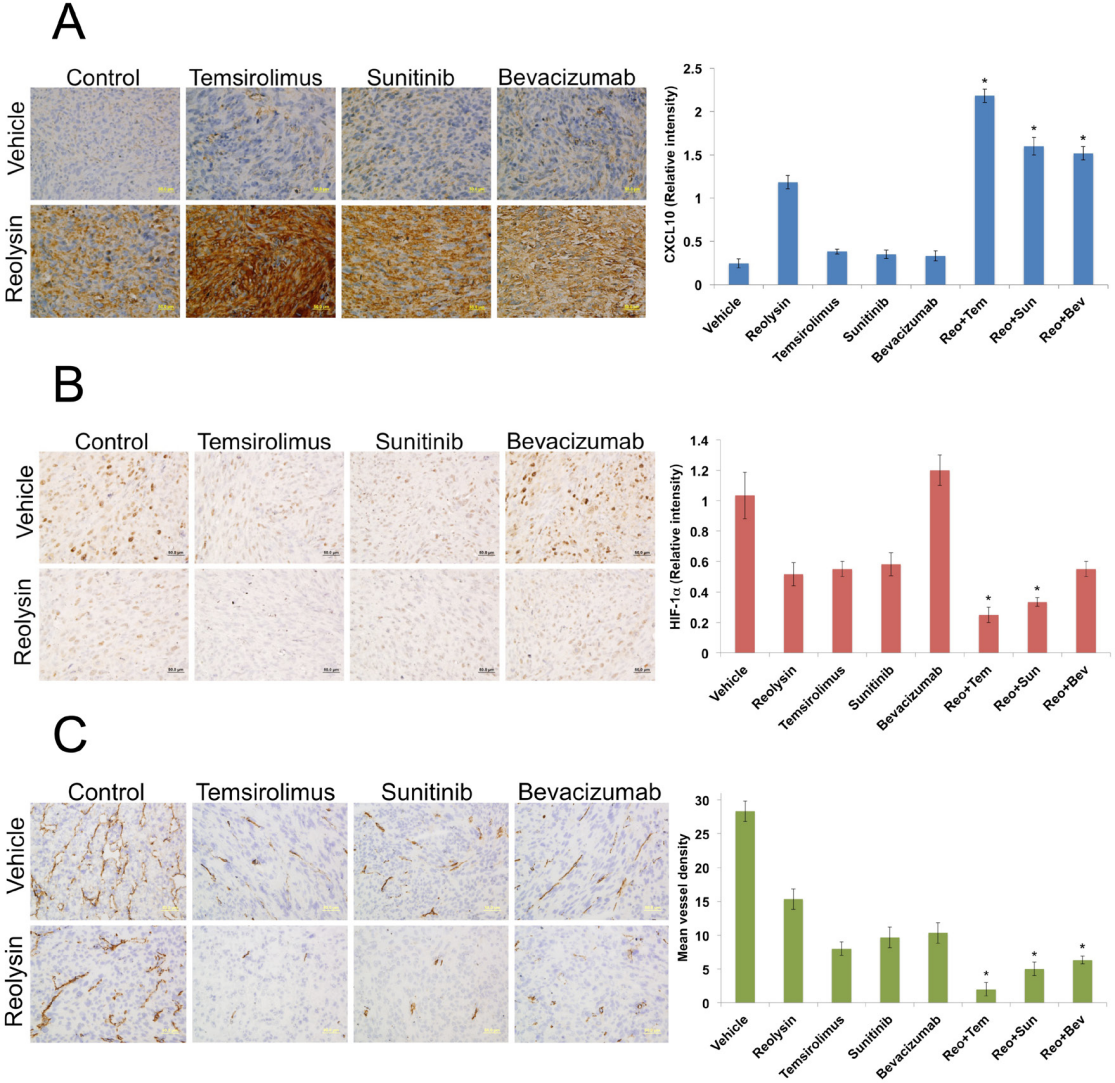
Reolysin inhibits angiogenesis *in vivo* **(A)** Reolysin increases CXCL10 expression, which is augmented by combination with anti-angiogenic therapies. CXCL10 levels were measured by IHC and staining intensity was quantified using ImageJ software. **(B)** Reolysin decreases HIF-1ɑ expression. IHC was used to measure HIF-1ɑ expression and staining intensity was determined by ImageJ software. **(C)** Reolysin inhibits angiogenesis. Mean vessel density was measured by CD31 staining. Quantification of mean vessel density was performed by manual counting of 5 random fields. *Indicates a significant difference compared single agent treatments, *p* < 0.05.

To further investigate the anti-angiogenic effects of Reolysin, we evaluated the activity of Reolysin in combination with FDA approved agents known to inhibit angiogenesis including the multi-tyrosine kinase inhibitor sunitinib, the anti-VEGF-A antibody bevacizumab, and the mTOR inhibitor temsirolimus. Interestingly, co-administration of these agents with Reolysin led to significantly superior induction of CXCL10 (Figure 5A) over what was achieved by any monotherapy evaluated. This suggests that Reolysin could potentially be used as a precision agent to augment and maximize the specific anti-angiogenic effects of temsirolimus, bevacizumab, and sunitinib in a manner that leads to improved overall anticancer activity. We next investigated the effects of Reolysin alone and in combination with these three agents in the SK-LMS-1 sarcoma xenograft model. Single agent therapy with Reolysin (5 x 10^8^ TCID_50_ IV, Q7Dx3), temsirolimus (5 mg/kg IV, [QDx5]x3), sunitinib (40 mg/kg PO, QDx21), and bevacizumab (10 mg/kg IP, Q3Dx6) all significantly reduced SK-LMS-1 tumor burden (Figure 5B). Importantly, Reolysin treatment significantly augmented the anticancer activity of all of the standard of care agents with the lowest tumor burden observed with the Reolysin and temsirolimus combination (Figure 5B). In addition, all combination therapies were very well tolerated with no visible signs of distress or animal weight loss (Figure 5C). Consistent with our *in vitro* data, immunohistochemical analysis of SK-LMS-1 tumors collected at study endpoint revealed a significant increase in CXCL10 levels following treatment with Reolysin (Figure 6A). The increase in CXCL10 expression was significantly more pronounced in each combination treatment with the most dramatic increase observed following exposure to Reolysin and temsirolimus. In addition to CXCL10 induction, HIF-1ɑ expression was also significantly decreased in all treatment groups except for bevacizumab (Figure 6B). This is consistent with prior reports showing that HIF-1ɑ levels increase following bevacizumab treatment due to a feedback response to VEGF-A signaling inhibition. Analysis of microvessel density demonstrated that Reolysin decreased vessel number in a manner that was similar to all three approved agents (Figure 6C). In addition, Reolysin administration also augmented the anti-angiogenic activity of bevacizumab, temsirolimus, and sunitinib (Figure 6C). Taken together, these results demonstrate that Reolysin has significant anti-angiogenic activity and can enhance the anticancer efficacy of a diverse array of approved agents with anti-angiogenic properties.

### Reolysin augments the activity of temsirolimus in four sarcoma xenograft models

**Figure 7:**
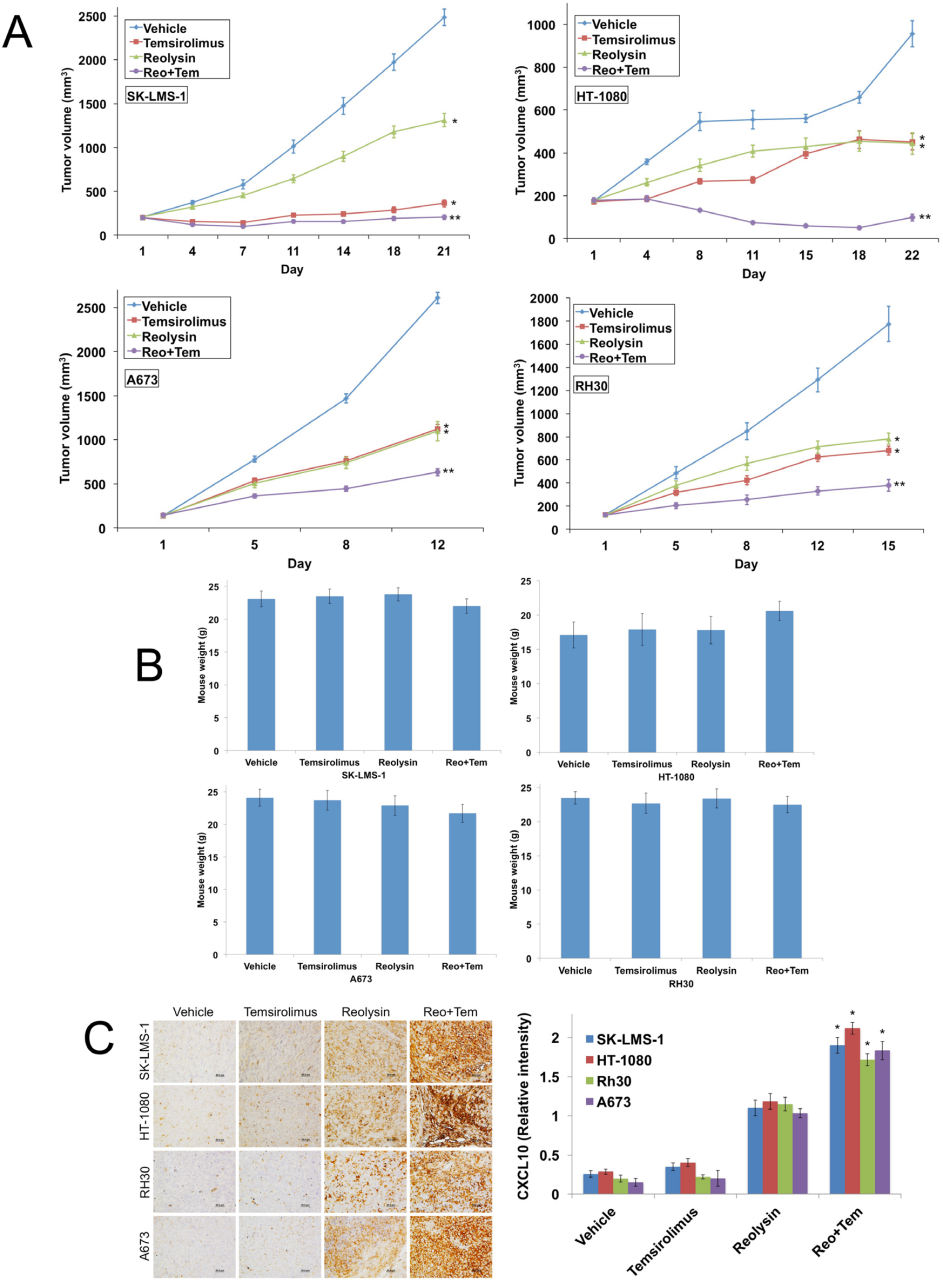
The combination of Reolysin and temsirolimus strongly reduces tumor burden in sarcoma xenograft models **(A)** SK-LMS-1, HT-1080, A673, and RH30 sarcoma cells (1 x 10^7^/mouse) were injected into the flanks of nude mice. When tumors reached approximately 150 mm^3^ in size, mice were randomized into groups and treated with 5 x 10^8^ TCID_50_ Reolysin Q7D, 5 mg/kg temsirolimus QD or both agents. Tumors were measured twice weekly. Mean ± SEM, *n* = 10. *Indicates a significant difference compared to vehicle or **either single agent treatment, *p* < 0.05. **(B)** Reolysin and temsirolimus are well tolerated *in vivo*. Animal body weight was determined at the end of the study to quantify drug-induced weight loss. Mean ± SD, *n* = 10. **(C)** Reolysin and temsirolimus increase CXCL10 expression. CXCL10 expression was measured by IHC and staining intensity was quantified using ImageJ software. Mean ± SD, *n* = 5. *Indicates a significant difference compared to single agent treatments, *p* < 0.05.

Our initial xenograft study in SK-LMS-1 revealed that Reolysin in combination with temsirolimus resulted in dramatically higher levels of CXCL10, increased angiogenesis inhibition, and significant antitumor efficacy. To further investigate the potential therapeutic benefit of the combination of Reolysin and temsirolimus, we conducted additional xenograft studies using 4 sarcoma models – SK-LMS-1, HT-1080, A673, and RH30. Tumor-bearing animals were randomized into treatment groups and administered either vehicle (PBS), 5 mg/kg temsirolimus IV (QDx5)x3, 5 x 10^8^ TCID_50_ Reolysin IV Q7Dx3, or both agents for 3 weeks or until a high tumor burden was reached in the vehicle treated mice. Treatment with either single agent significantly antagonized tumor progression (Figure 7A). However, the Reolysin and temsirolimus combination led to a decrease in tumor burden that was superior to either monotherapy (Figure 7A) in all 4 xenograft models. Importantly, the Reolysin and temsirolimus combination was well tolerated as no significant animal weight loss was observed at the completion of each study (Figure 7B). In agreement with our related *in vitro* assays, immunohistochemical analysis of tumor sections revealed a significant increase in CXCL10 expression in Reolysin exposed tumors, which was further augmented by the addition of temsirolimus (Figure 7C). Collectively, these data provide evidence that Reolysin possesses significant anti-angiogenic activity in sarcoma models irrespective of RAS status, inhibits HIF activity, dramatically increases CXCL10 expression, and improves the efficacy of agents that disrupt angiogenesis, such as temsirolimus.

## DISCUSSION

Reolysin is a clinical formulation of oncolytic reovirus that has the ability to selectively replicate in cells with an activated RAS pathway [2, 3]. This has led to its evaluation in patients with a variety of tumor types with a high prevalence of constitutive RAS signaling due to activating *RAS* point mutations or mutations in upstream pathway regulators such as epidermal growth factor receptor (EGFR) [20, 21]. The initial reports of reovirus hypersensitivity in RAS-driven malignancies likely reduced the priority for its intensive investigation as a cancer therapeutic in tumors types where RAS activation is less prevalent, such as soft tissue sarcomas [22–24]. At first look, our sarcoma data is supportive of this theory and consistent with data that we and other investigators have obtained in other tumor types in that the reovirus replication and *in vitro* anticancer efficacy was most pronounced in the mutant RAS HT-1080 fibrosarcoma cell line [2, 3, 8, 25, 26]. Minimal reovirus replication and apoptosis was observed in the WT RAS cell lines SK-LMS-1, A673, and RH30. However, a more detailed analysis revealed that activated RAS is not strictly required for the anti-sarcoma effects of reovirus. Indeed, gene expression analyses on the RAS mutant and sensitive HT-1080 and WT RAS and insensitive SK-LMS-1 sarcoma cell lines following *in vitro* reovirus infection showed that the anti-angiogenic factor CXCL10 was one of the most significant and dramatically upregulated genes in both sarcoma cell lines. CXCL10 has been reported to be a potent inhibitor of angiogenesis, tumor cell proliferation, and metastasis [11-13, 15-17]. It has emerged as a very exciting target for therapeutic stimulation within the context of cancer due to its important role in immune response and inflammation. In support of this concept, increased CXCL10 expression has been associated with improved response to immunotherapy [14, 27, 28]. Consistent with this role, we demonstrate that CXCL10 and reovirus inhibit VEGF-induced endothelial cell tube formation. Collectively, these data show that Reolysin treatment has significant anti-angiogenic activity through upregulation of CXCL10 expression. This particular aspect of its mechanism of action could also be harnessed to improve the efficacy of conventional chemotherapy.

Viral infection results in the secretion of IFNs, which promotes upregulation of CXCL10. A recent report demonstrated that IFNs and CXCL10 contribute to improved chemotherapeutic responses in neoplastic cells [27]. These data suggest that Reolysin treatment may be a viable approach to produce sustained CXCL10 expression and enhance the anticancer activity of conventional chemotherapy. Our data also demonstrate that cells with low reovirus infectivity (SK-LMS-1) induce CXCL10 to an equivalent extent to those that are more susceptible to reovirus infection (HT-1080). Numerous preclinical and clinical studies demonstrate the Reolysin is best utilized in combination with approved targeted agents or cytotoxic therapy [21, 25, 29–35]. Based on the effects of Reolysin on HIFs and CXCL10, we focused on evaluating Reolysin in combination with three approved agents known to exhibit anti-angiogenic properties as part of their mechanisms of action – temsirolimus, sunitinib, and bevacizumab. Importantly, Reolysin was able to significantly augment the anticancer activity of all 3 agents in the WT RAS SK-LMS-1 sarcoma xenograft model. Interestingly, combination treatment produced a further significant increase in CXCL10 expression and greater inhibition of angiogenesis compared to Reolysin monotherapy with the most dramatic increase observed with temsirolimus. Further investigation of Reolysin in combination with temsirolimus revealed enhanced CXCL10 upregulation and significant antitumor efficacy *in vivo* in all models tested, including 3 WT RAS sarcoma xenografts.

Additional studies of the effects of Reolysin on angiogenesis revealed that it potently antagonizes HIF-1ɑ activity. This is perhaps one of the most underappreciated aspects of its anticancer mechanism of action given that HIF-1ɑ induces the expression of many genes involved with angiogenesis, invasiveness, glycolysis, and drug resistance [18, 36, 37]. In addition to upregulation of CXCL10, reovirus also inhibited basal HIF-1ɑ activity and hypoxia-induced HIF-1ɑ activity using CoCl_2_, which mimics hypoxia by inhibiting PHD2 hydroxylation of HIF-1ɑ [38]. The effects of reovirus on decreasing HIF-1ɑ levels are consistent with prior reports in other model systems suggesting that the ability of reovirus to inhibit angiogenesis is not tumor specific [39–41]. Downregulation of HIF-1ɑ was reported to occur via proteasomal-mediated degradation and was dependent on the expression of the receptor for activated kinase C (RACK1) protein [40]. While this report links reovirus to HIF-1ɑ reduction, they did not investigate the ability of reovirus to inhibit angiogenesis *in vivo* or evaluate its potential to improve the efficacy of standard agents that target the tumor vasculature. Our study represents the first report of Reolysin’s ability to induce CXCL10 and effectively disrupt tumor angiogenesis *in vivo*. This important property is mutant *RAS*-independent, reovirus replication-independent, and opens up broad opportunities for this well tolerated agent to be used more effectively in combination with FDA approved conventional and targeted anticancer therapies for the treatment of highly angiogenic malignancies. Combination therapy clinical trials of Reolysin with temsirolimus or other agents that inhibit angiogenesis are warranted for the treatment of malignancies that exhibit high vascularity.

## MATERIALS AND METHODS

### Animals and cell lines

HT-1080 (fibrosarcoma), SK-LMS-1 (leiomyosarcoma), and A673 (Ewing’s sarcoma) cell lines were obtained from the American Type Culture Collection (ATCC) (Rockville, MD). RH30 (rhabdomyosarcoma) cells were purchased from the DSMZ (Braunschweig, Germany). All sarcoma cell lines were maintained in RPMI supplemented with 10% fetal bovine serum (FBS). Female nude mice (BALB/c background) were purchased from Harlan (Indianapolis, IN). Human umbilical vein endothelial cells (HUVEC) were obtained from ATCC. HUVECs were maintained in endothelial cell growth medium M200 (Invitrogen) with 10% FBS.

### Antibodies and chemicals

Antibodies were obtained from the following commercial sources: anti-tubulin (Sigma, St. Louis, MO), anti-GLUT1 (Cell Signaling, Beverly, MA), anti-HIF-1ɑ and anti-HIF-2ɑ (Novus Biologicals, Littleton, CO), anti-CXCL10 (Abcam, Cambridge, MA for IHC; R&D Systems, Minneapolis, MN for neutralization studies), anti-CD31 (BD Biosciences, San Jose, CA), rat anti-mouse IgG2a-HRP (Serotec, Raleigh, NC), and sheep anti-mouse-HRP and donkey anti-rabbit-HRP (Amersham, Pittsburgh, PA). Anti-reovirus antibody and Reolysin were kindly provided by Oncolytics Biotech, Inc. (Calgary, AB, Canada). Alexa Fluor 488 rabbit anti-goat was obtained from Molecular Probes (Eugene, OR). Sunitinib, bevacizumab, and temsirolimus were purchased from the hospital pharmacy.

### Quantification of drug-induced cytotoxicity

Cell viability was assessed by 3-(4,5-dimethylthiazol-2-yl)-2,5-diphenyltetrazolium bromide (MTT) assay. Cells were cultured in 96-well plates at a density of 10,000 cells per well and were treated with Reolysin for 72 h. Following drug treatment, MTT was added and viability was quantified using a microplate reader. The pro-apoptotic effects of Reolysin were quantified by propidium iodide (PI) staining and fluorescence activated cell sorting (FACS) analysis of sub-G_0_/G_1_ DNA and quantification of active caspase-3 positive cells by flow cytometry using a commercial kit (BD Biosciences, San Jose, CA) as previously described [42].

### Immunocytochemistry

Sarcoma cells were plated on chamber slides prior to oncolytic reovirus infection. Cells were fixed with 4% paraformaldehyde, permeabilized using 0.2% triton-X-100, and incubated overnight with anti-reovirus antibody. Fluorescent secondary antibodies were used to visualize protein localization. Images were captured using an Olympus fluorescent microscope (Center Valley, PA) with a DP71 camera and a 40X objective.

### Transmission electron microscopy

Cells were treated with 30 PFU/cell Reolysin for 48 h and processed for electron microscopy. Sections were cut in an LKB Ultracut microtome (Leica, Deerfield, IL), stained with uranyl acetate and lead citrate, and examined in a JEM 1230 transmission electron microscope (JEOL, USA, Inc., Peabody, MA). The percentage of reovirus positive cells were determined by counting 500 cells in random fields using an electron microscope. The fraction of cytoplasm containing containing reoviral particles was quantified using ImageJ software.

### RAS sequencing

DNA from sarcoma cells was isolated using the DNeasy mini kit (Qiagen Inc., Valencia, CA). DNA was eluted with 100 μl nuclease-free water and samples were checked for concentration and quality using a NanoDrop spectrophotometer. PCR amplifications were conducted using optimized cycling conditions per gene-exon. All samples were sequenced with forward and reverse primers spanning all exons to obtain the complete sequence of each gene. Sequencing reactions were run on an ABI 3130xl at the Nucleic Acid Core Facility.

### Expression microarrays

HT-1080 and SK-LMS-1 sarcoma cells were treated with 30 PFU/cell Reolysin for 48 h. Following drug treatment, total RNAs were isolated using the RNeasy Plus Mini Kit (Qiagen) and treated with TURBO DNA-free™ Kit (Applied Biosystems). 300 ng of total RNA per sample was amplified and hybridized to GeneChip^®^ Human Gene 1.0 ST arrays (Affymetrix, Inc.) according to the manufacturer’s instructions. Affymetrix CEL files were imported into Partek^®^ Genomics Suite™ 6.4 (Partek Inc.) using the default Partek normalization parameters and the robust multi-array average (RMA) analysis adjusted for probe sequence and GC content (GCRMA). Data normalization was performed across all arrays using quantile normalization.

### Quantitative real time polymerase chain reaction

cDNA from Reolysin treated cells were used for relative quantification by RT–PCR analyses. cDNA synthesis was performed from 1 μg RNA in a 20 μl reaction mixture using the high-capacity cDNA Reverse Transcription Kit (Applied Biosystems, Foster City, CA). *CXCL10, CXCL11*, and *GAPDH* transcripts were amplified using commercially available TaqMan^®^ Gene expression assays (Applied Biosystems, Foster City, CA). Relative gene expression was calculated with the 2^–ΔΔCt^ method using *GAPDH* as a housekeeping gene.

### Endothelial cell tube formation assay

The Endothelial Tube Formation Assay (Cell Biolabs Inc., San Diego, CA) was used to evaluate angiogenesis *in vitro*. Extracellular matrix (ECM) gel was thawed at 4 °C, coated on to a plate, and polymerized at 37 °C. HUVEC cells were treated with 30 PFU/cell Reolysin, 200 ng/ml recombinant CXCL10, and 2 μg/ml anti-CXCL10 neutralizing antibody with and without stimulation with VEGF (100 ng/ml) for 24 h. Endothelial tubes were quantified using a fluorescent microscope after staining with Calcein AM. Tube forming ability was determined by counting the total number of cell branches from 5 random fields.

### Immunoblotting

Cell pellets were harvested and lysed using Triton-X-100 lysis buffer (1% triton X-100, 150 mM NaCl, 25 mM Tris pH 7.5). Approximately 50 μg of total cellular protein from each sample were subjected to SDS-PAGE, proteins were transferred to nitrocellulose membranes, and the membranes were blocked with 5% nonfat milk in a Tris-buffered saline solution containing 0.1% Tween-20 for 1 h. The blots were then probed overnight with relevant antibodies, washed, and probed with species-specific secondary antibodies coupled to horseradish peroxidase. Immunoreactive material was detected by enhanced chemiluminescence (Protein Simple, Santa Clara, CA). Densitometry analysis to quantify band intensity was performed using an Alpha Innotech FluorChem HD2 gel documentation system (Alpha Innotech, Santa Clara, CA).

### Measurement of HIF-1ɑ activity

HIF-1ɑ activitywas determined by using the X-MAN HIF1A NanoLuc reporter kit of HCT116 cells (Horizon Discovery, Waterbeach, Cambridge). Cells were plated in 96 well plates with or without 100 μM CoCl_2_ to stimulate hypoxia and treated with increasing concentration of Reolysin for 24 and 48 h. Following treatment, Nano-Glo luciferase reagent was added to each well and luminescence was measured on a Molecular Devices (Sunnyvale, CA) plate reader.

### Quantification of VEGF and CXCL10 secretion by ELISA

To evaluate CXCL10 expression following treatment with Reolysin, sarcoma cells were plated in 6-well plates and treated with 30 PFU/cell Reolysin for 24 and 48 h. For VEGF measurements, cells were treated with 30 PFU/cell Reolysin with or without 100 μM CoCl_2_ for 6, 24, and 48 hours. Supernatants were collected and CXCL10 or VEGF protein levels were determined using Quantikine ELISA kits (R&D Systems, Inc., Minneapolis, MN). Cell numbers were equivalent in control and treated samples.

### Implantation of tumor cells and treatment schedule

Sarcoma cells were harvested from culture flasks and transferred to serum-free HBSS. Tumor cells (1 x 10^7^ cells) were injected into the right flank of female nude mice and allowed to establish tumors. Following tumor formation, animals were pair-matched by tumor size and placed into groups of 10 mice. Animals were then treated by IV injection of 5 x 10^8^ TCID_50_ Reolysin once a week (Q7D), 5 mg/kg temsirolimus IV daily (QDx5), 40 mg/kg sunitinib PO daily, 10 mg/kg bevacizumab IP twice a week (Q3D) or drug combinations. Tumor volume and animal weight measurements were recorded twice weekly. Tumor tissue was collected for immunohistochemistry (IHC) at the end of the study.

### Immunohistochemistry

Paraffin-embedded tumor sections were deparaffinized in xylene, a graded series of alcohol, and rehydrated in PBS. Heat-induced epitope retrieval on paraffin-embedded sections was performed by microwaving slides in a citrate buffer for 5 min. Frozen tumor sections were stained with CD31 antibody. Endogenous peroxides were blocked with a 3% hydrogen peroxide solution for 10 min. Slides were placed in a protein block solution (5% horse and 1% goat serum in PBS) for 20 min. followed by incubation with CXCL10, HIF-1ɑ, and CD31 antibodies at 4°C overnight. After washing with PBS, slides were incubated in the appropriate HRP-conjugated secondary antibodies for 1 hour at ambient temperature. Positive reactions were visualized using 3,3'-diaminobenzidine diaminobenzidine (Dako, Carpinteria, CA) for 10 min. The slides were rinsed with water and counterstained with Gill's hematoxylin (Sigma, St. Louis, MO). Images were captured using an Olympus fluorescent microscope (Center Valley, PA) with a DP71 camera and a 20X objective. Image-Pro Plus software Version 6.2.1 (MediaCybernetics, Bethesda, MD) was used for image acquisition. ImageJ software was used for quantification of CXCL10 and HIF-1ɑ levels by densitometric analysis of five random fields containing viable tumor cells. Quantification of mean vessel density was conducted by counting the number of vessels in five random fields.

### Statistical analyses

Statistical significance of differences observed between samples was determined using the Tukey-Kramer Comparison Test or the Student’s *t* test. Differences were considered significant in all experiments at p < 0.05.

## SUPPLEMENTARY MATERIALS TABLES






