# Supplementary material for: Oncolytic reovirus inhibits angiogenesis through induction of CXCL10/IP-10 and abrogation of HIF activity in soft tissue sarcomas
Source: Oncotarget. 2017 Sep 30;8(49):86769–83. doi: 10.18632/oncotarget.21423 (PMC5689724; doi:10.18632/oncotarget.21423)
Supplement: Supplementary file 1 [file oncotarget-08-86769-s001.pdf]

Oncolytic reovirus inhibits angiogenesis through induction of CXCL10/IP-10 and abrogation of HIF activity in soft tissue sarcomas

SUPPLEMENTARY MATERIALS

Supplementary Table 1: RAS mutations in sarcoma cell lines

Cell Line	N-RAS	nt Change
HT-1080	Q61K	CAA > AAA
A673	WT	N/A
RH30	WT	N/A
SK-LMS-1	WT	N/A

Supplementary Table 2: Up-regulated genes following Reovirus treatment HT-1080 genes up-regulated following Reovirus treatment

See Supplementary File 1

Supplementary Table 3: Up-regulated genes following Reovirus treatment SK-LMS-1 genes up-regulated following Reovirus treatment

See Supplementary File 2
